# Retinal Development and Pathophysiology in Kcnj13 Knockout Mice

**DOI:** 10.3389/fcell.2021.810020

**Published:** 2022-01-12

**Authors:** Xiaodong Jiao, Zhiwei Ma, Jingqi Lei, Pinghu Liu, Xiaoyu Cai, Pawan K. Shahi, Chi-Chao Chan, Robert Fariss, Bikash R. Pattnaik, Lijin Dong, J. Fielding Hejtmancik

**Affiliations:** ^1^ Ophthalmic Genetics and Visual Function Branch, National Eye Institute, National Institutes of Health, Bethesda, MD, United States; ^2^ Genetic Engineering Core, National Eye Institute, National Institute of Health, Bethesda, MD, United States; ^3^ Departments of Pediatrics and Ophthalmology and Visual Sciences and the McPherson Eye Research Institute, University of Wisconsin, Madison, AL, United States; ^4^ Imaging Core, National Eye Institute, National Institutes of Health, Bethesda, MD, United States

**Keywords:** retina, retinal dystrophy, snowflake vitreoretinal dystrophy, kcnj13/kir7.1, retinal degeneration

## Abstract

**Purpose:** We constructed and characterized knockout and conditional knockout mice for KCNJ13, encoding the inwardly rectifying K^+^ channel of the Kir superfamily Kir7.1, mutations in which cause both Snowflake Vitreoretinal Degeneration (SVD) and Retinitis pigmentosa (RP) to further elucidate the pathology of this disease and to develop a potential model system for gene therapy trials.

**Methods:** A Kcnj13 knockout mouse line was constructed by inserting a gene trap cassette expressing beta-galactosidase flanked by FRT sites in intron 1 with LoxP sites flanking exon two and converted to a conditional knockout by FLP recombination followed by crossing with C57BL/6J mice having Cre driven by the VMD2 promoter. Lentiviral replacement of Kcnj13 was driven by the EF1a or VMD2 promoters.

**Results:** Blue-Gal expression is evident in E12.5 brain ventricular choroid plexus, lens, neural retina layer, and anterior RPE. In the adult eye expression is seen in the ciliary body, RPE and choroid. Adult conditional Kcnj13 ko mice show loss of photoreceptors in the outer nuclear layer, inner nuclear layer thinning with loss of bipolar cells, and thinning and disruption of the outer plexiform layer, correlating with Cre expression in the overlying RPE which, although preserved, shows morphological disruption. Fundoscopy and OCT show signs of retinal degeneration consistent with the histology, and photopic and scotopic ERGs are decreased in amplitude or extinguished. Lentiviral based replacement of Kcnj13 resulted in increased ERG c- but not a- or b- wave amplitudes.

**Conclusion:** Ocular KCNJ13 expression starts in the choroid, lens, ciliary body, and anterior retina, while later expression centers on the RPE with no/lower expression in the neuroretina. Although KCNJ13 expression is not required for survival of the RPE, it is necessary for RPE maintenance of the photoreceptors, and loss of the photoreceptor, outer plexiform, and outer nuclear layers occur in adult KCNJ13 cKO mice, concomitant with decreased amplitude and eventual extinguishing of the ERG and signs of retinitis pigmentosa on fundoscopy and OCT. Kcnj13 replacement resulting in recovery of the ERG c- but not a- and b-waves is consistent with the degree of photoreceptor degeneration seen on histology.

## Introduction

Mutations in the inwardly rectifying potassium channel gene KCNJ13 have been shown to cause both snowflake vitreoretinal degeneration (SVD) (Hejtmancik et al., 2008) and Leber congenital amaurosis (LCA) (Sergouniotis et al., 2011). SVD, first described in 1974 by Hirose et al. (1974), is a vitreoretinal degeneration characterized by corneal guttae, cataract, fibrillar vitreous degeneration, and peripheral retinal abnormalities including characteristic small yellow-white dots, and sheathing followed by disappearance of retinal vessels, chorioretinal pigmentation and atrophy at later stages (Lee et al., 2003). LCA, the most severe form of retinal degeneration, shows severe visual impairment and retinal dysfunction in the first year of life, with night blindness and constricted visual fields often accompanied by nystagmus. While these retinal diseases have some overlapping features, they are distinguished by the relatively preserved vision and the higher frequency of vitreal changes in SVD. It appears that the autosomal dominant inheritance pattern of SVD due to the Kir7.1 R162W mutation probably results from depolarization of the resting membrane potential of the RPE by the mutant protein, perhaps exacerbated by insertion of R162W mutant Kir7.1 molecules into the tetrameric Kir7.1 channels in a partial dominant negative effect (Hejtmancik et al., 2008; Pattnaik et al., 2013; Zhang et al., 2013); while the autosomal recessive LCA phenotype results from a complete loss of channel function (Sergouniotis et al., 2011; Pattnaik et al., 2015; Perez-Roustit et al., 2017).

The Kcnj13 gene encodes Kir7.1, a K^+^ channel belonging to the Kir family, a group of inwardly rectifying K^+^-transport channels. Kir7.1, first described in 1998 (Krapivinsky et al., 1998), shows marked differences in both sequence and functional properties from other members of the Kir channel superfamily (Krapivinsky et al., 1998; Shimura et al., 2001). Kir 7.1 channels show low single channel conductance and low sensitivity to external Ba^2+^ blockade and are relatively independent of voltage and K+ concentration, whereas other members of the Kir family, which present strong inward rectification properties, primarily provide currents involving K^+^ influx into cells (Hughes and Takahira, 1996; Krapivinsky et al., 1998; Shimura et al., 2001). At hyperpolarized membrane potentials, Kir7.1 exhibits a large inward K^+^ current, but at physiological membrane potentials, the channel facilitates the efflux of intracellular K^+^ (Krapivinsky et al., 1998; Shimura et al., 2001). Among other tissues including hypothalamic neurons in which it is regulated by the melanocortin-4-receptor (Ghamari-Langroudi et al., 2015), brain choroid plexus, lung, renal and intestinal epithelia and thyroid follicular cells (Döring et al., 1998; Ookata et al., 2000; Cornejo et al., 2018), Kir7.1 is present in the apical membrane of retinal pigmented epithelium (RPE) and the choroid, in which the Na^+^/K^+^-pump is also expressed apically (Hughes and Takahira, 1996; Shimura et al., 2001). Here, it is regulated by membrane phospholipids including phosphatidylinositol 4,5-biphosphate which is cleaved upon binding of oxytocin by the oxytocin receptor, a G protein-coupled receptor (Pattnaik and Hughes, 2009; York et al., 2017).

Since mutations in KCNJ13 were identified as a cause of SVD and LCA a number of approaches and model systems have been applied to verify causality and dissect the mechanisms and pathogenesis of these diseases. Mice with a loss-of-function Kcnj13 mutation were generated by N-ethyl-N-nitrosourea (ENU) mutagenesis and homozygotes were found to have neonatal lethal tracheal and smooth muscle defects (Yin et al., 2018). Similarly, mice in which the Kcnj13 gene locus was deleted were generated using VelociGene (Valenzuela et al., 2003) and found to die of respiratory and palatal deformities in the first day of life (Villanueva et al., 2015). Zhong et al. took advantage of mosaicism in CRISPR-mediated genomic indels of Kcnj13 in mice and were able to show that while RPE cells lacking Kir7.1 survived the underlying photoreceptors degenerated but could be rescued by nearby wild type or heterozygous RPE cells with an intact Kcnj13 gene (Zhong et al., 2015). They later used a Best1-cre conditional knockout of Kcnj13 in the retina, observing thinning of the outer nuclear layer and reduced light responses in mice with a high percentage of cells lacking the Kcnj13 gene (Roman et al., 2018) However, in this model Cre is expressed in only 10–90% of RPE cells, leading to patchy Kcnj13 expression in the conditional ko mouse retinas so that only 45% of the mice had a retinal phenotype. They were, however, able to overcome this in part by use of a TdTomato fluorescent indicator of Cre to select mice with high levels of Cre expression.

Here, we describe a Kcnj13 knockout mouse model with an initial ‘knockout first’ construct removing Kcnj13 universally with expression of beta-galactosidase as a marker followed by FLP recombination generating a conditional RPE specific conditional knockout using a VMD2-Cre mouse line. With these models we confirm the previous findings regarding Kcnj13 expression and the retinal phenotype of conditional ko mice, delineate early expression patterns of Kcnj13 in the mouse eye and brain and extend these to show complete loss of the photoreceptors including the outer nuclear layer, and outer plexiform layer accompanied by extinguished ERGs and fundus findings typical for retinal degeneration in mice with widespread absence of Kcnj13 expression. Finally, we show that replacement of Kcnj13 expression using recombinant Lentivirus can partially rescue potassium pump activity in the RPE and hence the ERG c-wave but not a- and b-wave amplitudes.

## Materials and Methods

### Generation of the Kcnj13 Knockout First Allele and Subsequent Conversion to Conditional Knockout Allele

**FIGURE 1:**
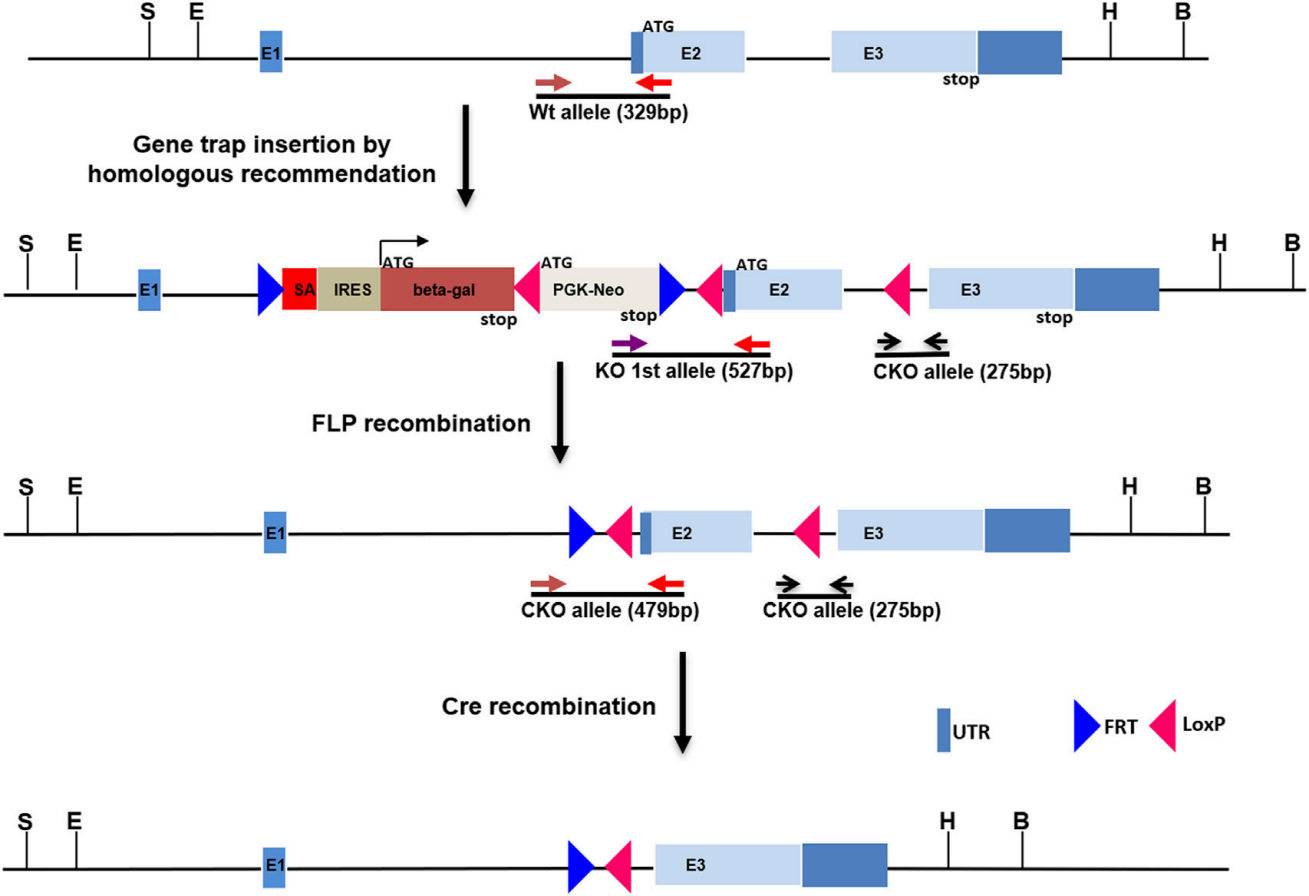
Construction of Kcnj13 knockout alleles. For the knockout first (KO 1^st)^ allele a cassette consisting of a splice acceptor followed by the beta-galactosidase gene, a LoxP site, and PGK-Neo and flanked by FRT sites is inserted into the first intron of the Kcnj13 gene and LoxP sites are inserted flanking Exon 2. The KO 1^st^ allele is converted to a conditional knockout allele by FLP recombination leaving LoxP sites flanking exon 2, critical for Kcnj13 function, for removal in the presence of Cre. Positions of genotyping primers are shown.

All animal procedures were approved by the Institutional Animal Care and Use Committee (IACUC) of the National Eye Institute and the University of Wisconsin- Madison. The Kcnj13 knockout (KO) first allele was constructed with classic homologous recombination in mouse R1 (SV129) embryonic stem (ES) cells by inserting an intron trap cassette consisting of a splice acceptor En2 followed by the *β*-galactosidase gene (PMID: 1592261) downstream of exon one of the Kcnj13 gene. LoxP sites were included as well flanking Exon two of the gene (Figure 1). Homologous recombination of the intron trap cassette at the target locus created a KO allele first which disrupted splicing of Kcnj13 mRNA by trapping upstream exon onto En2 splicing acceptor. As a result, the beta-gal coding sequence is rendered under the control of Kcnj13 locus and can be used as a reporter, tracking expression pattern of the target gene. Upon removal of the intron trap cassette by FLP recombination, the KO allele is converted to a conditional allele of Kcnj13 gene as diagrammed in Figure 1. Germline transmission was readily achieved and the F1 founders with the KO first allele were crossed back into the C57bl6/j background in several generations until they reached a congenic state. For postnatal studies the KO first allele was converted to a conditional RPE knockout allele by crossing to an RPE-specific VMD2-Cre line.

Genotyping of both KO first and conditional alleles were conducted by genomic PCR with primers listed in Supplementary Table S1.

### DNA Sequencing

Constructs and modifications to the Kcnj13 gene were amplified using primers shown in Supplementary Table S1. PCR reagents included: 10x PCR buffer: 1.0 ul, Mg2+ 0.6 ul, dNTP 0.5 ul, 10 pm primer 0.5 + 0.5 ul. Taq 1 u, DNA 40 ng, H2O up to 10 ul. Cycling including a touchdown PCR reaction for the first 15 cycles: 94 °C for 4 min, followed by decreasing the annealing temperature from an initial 64 °C in a stepwise fashion by 0.5 °C every second cycle. For the later 20 cycles: 94 °C for 40 sec, 57 °C for 30 sec and 72 °C for 1.5 min, and finally a prolonged elongation step at 72 °C for 10 min. PCR product was purified and analyzed by Sanger sequencing using an ABI 3130 sequencer with Big Dye Terminator Ready reaction mix according to the manufacturer’s instructions (Applied Biosystems, Foster City, CA). Sequencing results were analyzed using Mutation Surveyor v3.30 (Soft Genetics, State College, PA) or DNASTAR Navigator 17 (DNASTAR, Madison, WI).

### Histology

Mice were euthanized by CO_2_ inhalation after ERG data acquisition, and each eye was washed immediately with 1×PBS (Life Technologies, United States) and 10% neutral buffered formalin (Sigma-Aldrich, United States). The eyes were fixed for 48 h in formalin and processed for histological analysis. The tissue was embedded in paraffin, and 3 μm sections were prepared using a Microtome. The sections through the pupillary-optic nerve axis were deparaffinized, hydrated, and finally stained with Hematoxylin and Eosin (H&E). Five fields per section were selected for examination using an Olympus BX-51 microscope (Olympus, United States), while three sections (30 μm apart) from each eye (*n* = 3 mice per group) were used for analysis.

### Beta-Galactosidase Staining

β-galactosidase staining was performed by following an established protocol. For staining of mouse embryos, embryos of E11.5 to E15.5 were isolated in cold PBS, and switched to 4% paraformaldehyde (PFA) in 1x PBS on ice for 30 min and then washed in PBS for 5 min at room temperature. Embryos were incubated in *β*-galactosidase staining solution (1 mg/ml X-gal dissolved in dimethylformamide, 4 mM K4 Fe(CN)6.3H2O, 4 mM K3Fe(CN)6, 2 mM MgCl2) overnight at room temperature in the dark, washed 3 times for 15 min each in PBS, and further fixed in 2% paraformaldehyde with 2% glutaraldehyde.

β-Galactosidase staining of frozen sections was performed as follows. Embryos were pre-fixed in 4% PFA on ice for 30 min and embedded in OCT. Frozen sections were cut at 30–50 µm and placed on slides. After drying, slides were stained with the same *β*-galactosidase staining solution at 37°C for up to 30 min, washed in PBS for 5 min at room temperature, and mounted with mounting medium and cover slips and viewed under a Nikon microscope.

### Fundus Photography of Mouse Retinas

Funduscopic examinations of nine-month- old KCNJ13CKO ko/ko/ VMD2-Cre +/+ and KCNJ13CKO ko/ko/ VMD2-Cre −/− control mice (age and sex matched) were anesthetized with a mixture of xylazine (6 mg/kg) and ketamine (100 mg/kg), and pupils were dilated with a topical drop of tropicamide and phenylephrine. After pupil dilation, lubricant eye gel (Alcon) was applied to the cornea. Fundus images were captured by Micron III fundus camera (Phoenix Research Laboratories, Inc., Pleasanton, CA).

### Immunofluorescent Assessment of Mouse Retinas

Mouse eyes were fixed in 4% paraformaldehyde in 1x PBS for 2 h. After embedding in Optimal Cutting Temperature (OCT) (Tissue-Tek, Torrance, CA) with appropriate orientation to allow cutting of sagittal sections, the eye was cut into frozen sections of 10 µm thickness and cryosections were permeabilized with 0.3% Triton X-100 in PBS, the sections were blocked with blocking buffer (PBST, 5% BSA, 1% goat or donkey serum) for 1 h at room temperature. The sections were then incubated in a humidified chamber to prevent evaporation with primary antibodies overnight at room temperature. After three washes in PBST for 15 min each, sections were incubated with the corresponding secondary antibodies at room temperature for 1.5 h followed by three PBST washes and finally staining with DAPI (Sigma-Aldrich, (United States) for 15 min. Five fields per section were selected for examination using a confocal laser scanning

microscope (Zeiss LSM 700, Carl Zeiss Inc, Thornwood, NJ), while three sections (30 μm apart) from each eye (*n* = 3 mice per group) were used for analysis. Antibodies used were 1:200 mouse anti-Kir7.1 (C-12, Santa Cruz Biotechnology) and 1:200 rabbit anti-Cre (Novagen #69050-3, Millipore Sigma). Secondary antibodies 1:300 Alexa Fluor555 donkey anti-mouse and 680 donkey Anti-rabbit IgG. F-actin was labeled by incubating for 4 h with phalloidin1:500(Invitrogen) to enable visualization of RPE cell morphology. Rod bipolar cells were visualized with anti-PKCα (Invitrogen).

### Optical Coherence Tomography

OCT was performed using a Spectralis multi-modality diagnostic imaging system (Heidelberg Engineering). The system has a platform designed for easy orientation and aligning of mice for retinal imaging and provides a resolution of 2 μm. The mice were anesthetized by intraperitoneal injection with a mixed solution of ketamine and xylazine. The pupils were dilated with 1% tropicamide eye drops prior to imaging. Radial volume scan (centered on the optic disc, consisting of 100 B-scans) was acquired using image analysis software provided by Spectralis.

### Mouse Electroretinography

Electroretinography (ERG) responses were recorded for seven-month-old Kcnj13 Cre homozygous KO and control mice (age- and sex-matched) using an Espion E2 system (Diagnosys. Lowell, MA). Briefly, the mice were dark adapted for 12 h before the ERG examination. Prior to the scotopic examination, mice were anesthetized under red light by intraperitoneal injection of ketamine and xylazine. Additionally, Proparacaine hydrochloride ophthalmic solution (0.5%) was used to anesthetize the cornea while tropicamide (1%) and phenylephrine hydrochloride (2.5%) solutions were used to dilate the pupil. Flash ERG recordings were obtained simultaneously from both eyes using gold wire loop electrodes, with the reference electrode placed in the animal’s mouth and the ground subdermal electrode at the tail. ERG responses were obtained at increasing light intensities over the ranges of 1 × 10^–4^–10 cd s/m^2^ under dark-adapted conditions, and 0.3–100 cd s/m^2^ under a background light that saturates rod function. The stimulus intervals between flashes varied from 5 s at the lowest stimulus strengths to 60 s at the highest ones. Two to ten responses were averaged depending on flash intensity. ERG signals were sampled at 1 kHz and recorded with 0.3 Hz low-frequency and 300 Hz high-frequency cutoffs. For acquisition of c-wave, the eyes were flashed with light intensities of 25 cd s/m^2^ for 4 s. Analysis of a-wave and b-wave amplitudes was performed using a customized version of Espion ERG Data Analyzer software (v2.2) that digitally filters out high-frequency oscillatory potential wavelets. Statistical significance between two groups, was assessed by Student’s t-test. The level of significance was chosen to be *p* = 0.05 using GraphPad Prism 7 (GraphPad Software, San Diego, CA).

### Gene Therapy of Kcnj13 Ko Mouse

Gene therapy was performed on conditional knock out mice of either sex that showed no c-wave when ERG was screened at 4 weeks of age. The a- and b-waves of these mice were either completely absent or had a small amplitude. An intraperitoneal injection of a Ketamine (80 mg/kg)/Xylazine (16 mg/kg) cocktail was used to anesthetize these animals. The eyes were dilated with 1% tropicamide solution after being anesthetized with 0.5% proparacaine HCl. Before the injection, the mice were placed on a heating pad that was maintained at 37°C. The injections were carried out using UMP3 ultramicropump with a Nanofil syringe and RPE-KIT (World Precision Instruments, Sarasota, FL) while the mice were under the surgical microscope. These mice were injected with lentiviruses containing the Kir7.1 gene, which was tagged with GFP protein at the N-terminus and was either driven by the Ef1a promoter or the VMD2 promoter (VectorBuilder Inc., Chicago, IL). As a control, buffer was injected without the viral particles. The formation of the bleb was used to determine whether or not the injection was successful. The transduction of the RPE with lentivirus was confirmed by imaging RPE fluorescence using a Nikon C2 confocal microscope (Nikon Instruments Inc.) 4 weeks after the injection. ERGs were performed on the treated mice 1, 2, and 4 weeks after injection.

## Results

In order to investigate further the pathophysiological role of KCNJ13 mutations in snowflake vitreoretinal degeneration (SVD, MIM 193230) and Leber congenital amaurosis (LCA16, MIM 614186) a knockout mouse model was constructed by inserting a gene trap cassette in intron 1, which consisted of a splice accepter (SA) followed by IRES and beta galactosidase sequences. The gene trap cassette is flanked by FRT sites and the gene trap targeting vector is constructed so that exon two of the Kcnj13 gene is flanked by LoxP sites (Figure 1). This intron gene trap disrupted splicing of the target gene by forced splicing of the exon one transcript to the SA forming an exon1-SA fused transcript. Expression of beta-galactosidase driven by the Kcnj13 promoter was then be detected by Blue-gal assay. As has been reported (Villanueva et al., 2015), while heterozygous mice showed no obvious differences from wild type, no homozygous knockout mice were seen in any litters. However, while previous reports documented liveborn knockout pups that died shortly after birth (Villanueva et al., 2015; Zhong et al., 2015; Yin et al., 2018), in this study no homozygous ko mice were detected at or after E15.5 (Table 1), strongly suggesting that death occurred before E15.5 (*p* ≤ 0.0075).

**TABLE 1:** Survival of Kcnj13 knockout first and conditional knockout mice identified at increasing gestational ages and surviving to adulthood. No knockout first homozygous mice were identified after E15.5.

Genotype	Knockout first allele					Conditional allele
	adult	E18.5	E15.5	E14.5	E12.5	adult
	# pups (%)	# pups (%)	#pups (%)	#pups (%)	#pups (%)	#pups (%)
WT	95(43%)	6 (46%)	13 (52%)	1 (14%)	9 (32%)	3 (27%)
Heterozygous	128 (57%)	7 (54%)	12 (48%)	5 (72%)	11 (39%)	6 (55%)
Homozygous	0	0	0	1 (14%)	8 (29%)	1 (9%)^a^
Total	223	13	25	7	28	11

a While additional mouse litters were not genotyped for segregation analysis, a large number of KCNJ13CKO ko/ko, VMD2-Cre +/+ mice were bred for further functional and histological studies.

**FIGURE 2:**
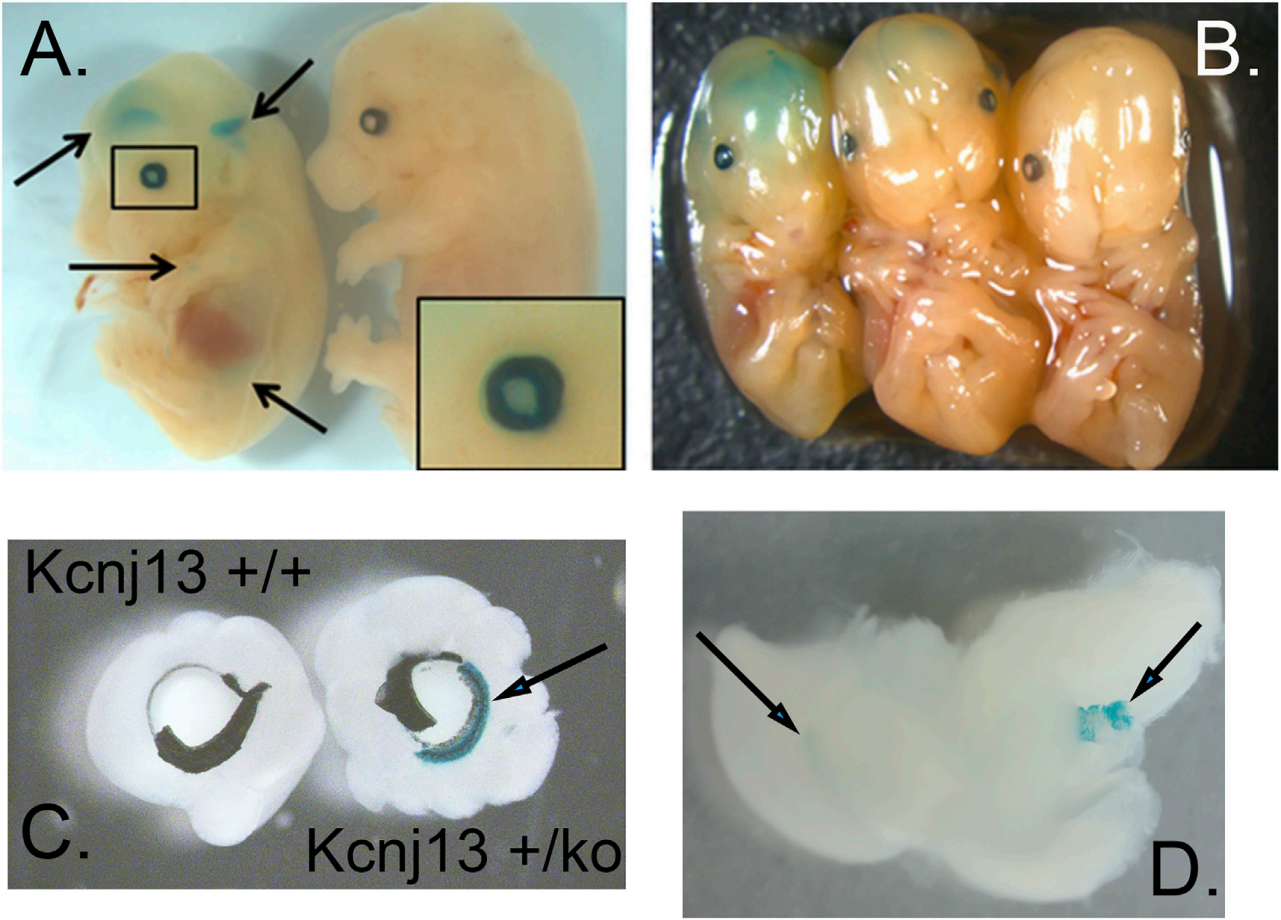
Expression of Kcnj13 as monitored by *β*-galactosidase expression from the ko1st allele in E13.5 mouse embryos. **(A,B)** Whole mount showing *β*-gal staining in cerebral ventricles and the eye. Arrows show the lateral and third ventricles as well as lower diffuse expression in other tissues, and the enlarged box shows expression in the eye, including the lens. Note that the homozygous embryo on the left is slightly smaller than the heterozygous [**(B)** **middle**] or wild type [**(A,B)** **right**] embryos. **(C)** Beta-gal staining of the anterior retina and ciliary body revealed after peeling of attached iris. **(D)** Beta-gal staining seen in the lateral ventricle (left arrow) and choroid plexus of the 4^th^ ventricle (right arrow).

**FIGURE 3:**
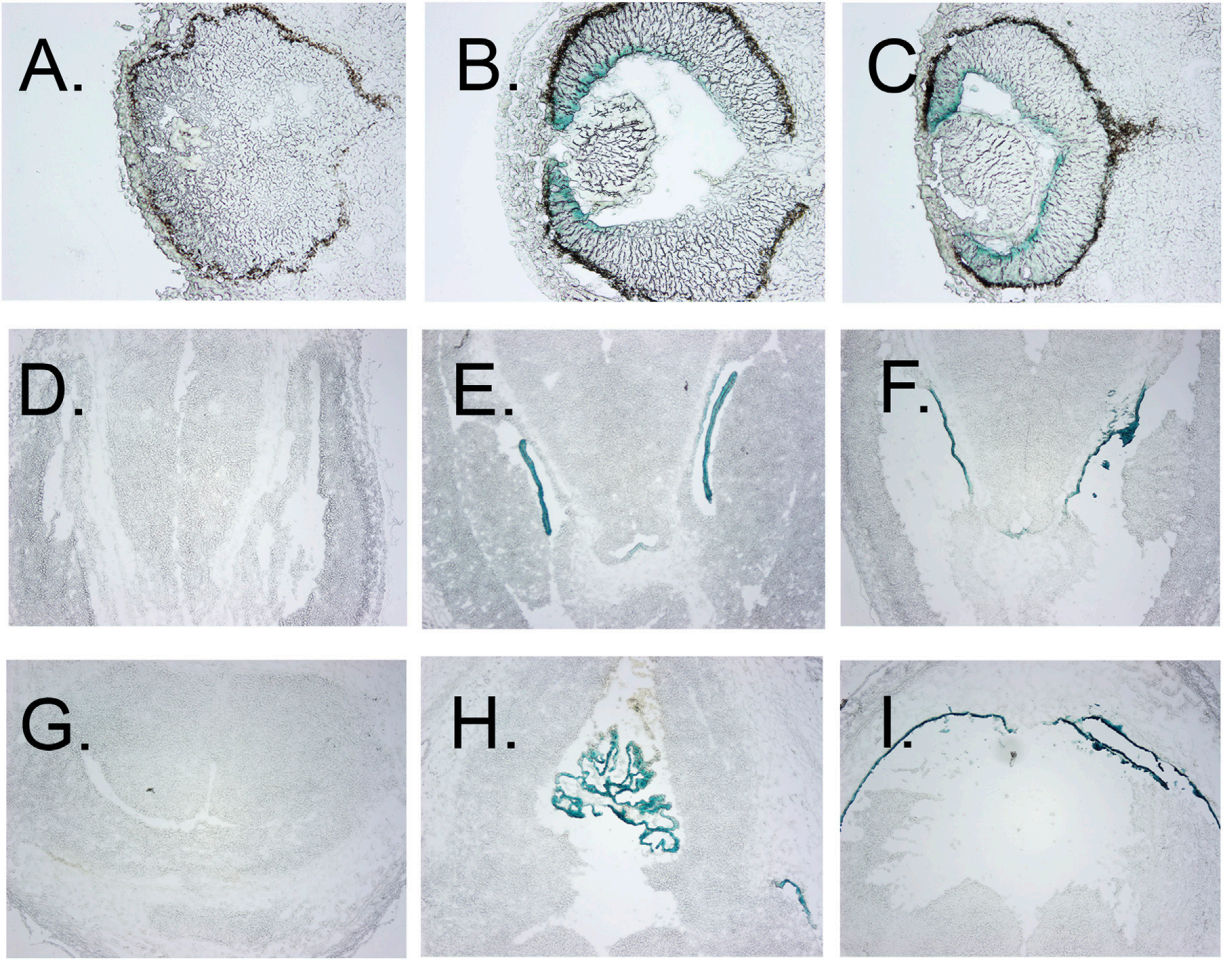
Localization of *β*-gal staining in tissue sections. **(A–C)** *β*-Gal staining of eyes of E12.5 wt, heterozygous, and homozygous KO1^st^ mice, respectively, showing staining of the anterior but not posterior retina, less in the lens primary nuclear fibers and even lighter staining in the anterior epithelia. **(D–F)** *β*-galactosidase staining of the choroid plexus of the lateral ventricles of E12.5 wt, heterozygous, and homozygous KO1^st^ mice, respectively. I-K. *β*-galactosidase staining of choroid plexus of the third ventricle of E12.5 wt, heterozygous, and homozygous KO1^st^ mice, respectively. showing staining in the areas of the primary nuclear fibers and lighter staining in the anterior epithelia. **(H)** Section through the lens of a wt control. **(I)** *β*-galactosidase staining of a section through the retina showing activity in the cells of the anterior but not posterior retina. J. Section through the retina of a wt control mouse.

**FIGURE 4:**
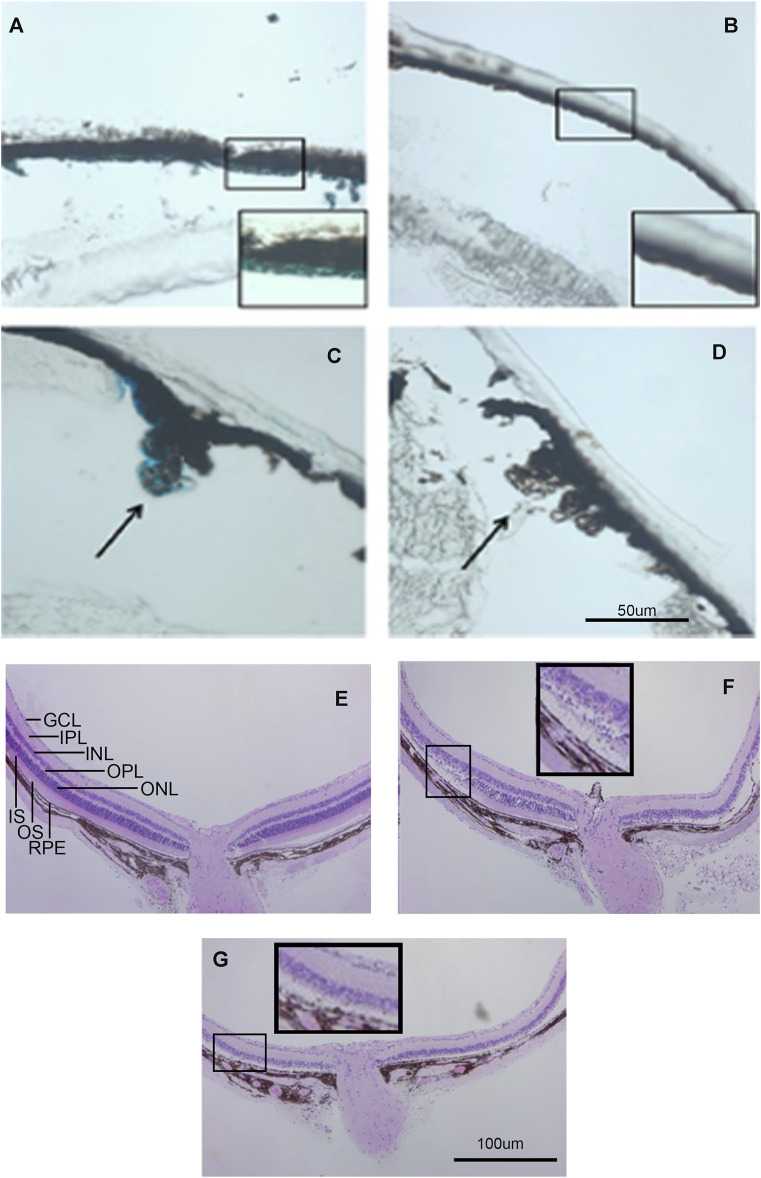
Kcnj13 expression in adult mice. **(A)** *β*-gal staining of adult heterozygous mouse retina showing positive staining in the RPE. **(B)** *β*-gal staining of a wt control retina. **(C)** *β*-gal staining of the ciliary body in an adult heterozygous mouse tissue section. **(D)** *β*-gal staining of a wt control retina. **(E)** H&E-stained retinal section from a 6-month-old KCNJ13CKO ko/ko, VMD2-Cre −/− mouse. **(F)** H&E-stained retinal section from a 6-month-old homozygous KCNJ13CKO ko/ko, VMD2-Cre +/+ mouse showing loss of photoreceptor cells on the right side and preservation of retinal morphology on the left near the optic nerve. **(G)** H&E-stained retinal section from a 6-month-old homozygous KCNJ13CKO ko/ko, VMD2-Cre +/+ mouse showing severe retinal degeneration throughout the retina.

Galactosidase staining was apparent in intact E12.5-day embryos in the eye lens, the choroidal plexus of the fourth and fifth ventricles of the brain, and the anterior retina (Figure 2). It increases by E15, being present to a lesser extent in other parts of the embryos. In addition, the Kcnj13 KO 1^st^ homozygous embryos were smaller than the heterozygous or homozygous wild type embryos at this stage. The localization of Kcnj13 expression at E 12.5 was confirmed by examination of tissue sections stained for *β*-galactosidase. Notably, Blue-gal staining was apparent in sections of the anterior retina near the presumptive ciliary body and especially those cells adjacent to the presumptive vitreous and choroid but not the posterior regions of the retina (Figures 3A–C). Staining is also prominent in the choroid plexus of the lateral, third, and fourth ventricles (Figures 3D–I), and the primary fiber cells and to a lesser extent the anterior epithelia of the lens (Figures 3A–C). Because the Kcnj13 KO is lethal before E15.5, *β*-galactosidase localization and staining of adult mice could only be carried out in heterozygotes (Figures 4A–D). These sections show strong *β*-galactosidase staining in the retinal pigment epithelial cells (RPE) across the retina, and the ciliary body but not in the photoreceptor layers adjacent to the RPE (Figures 4A,C).

**FIGURE 5:**
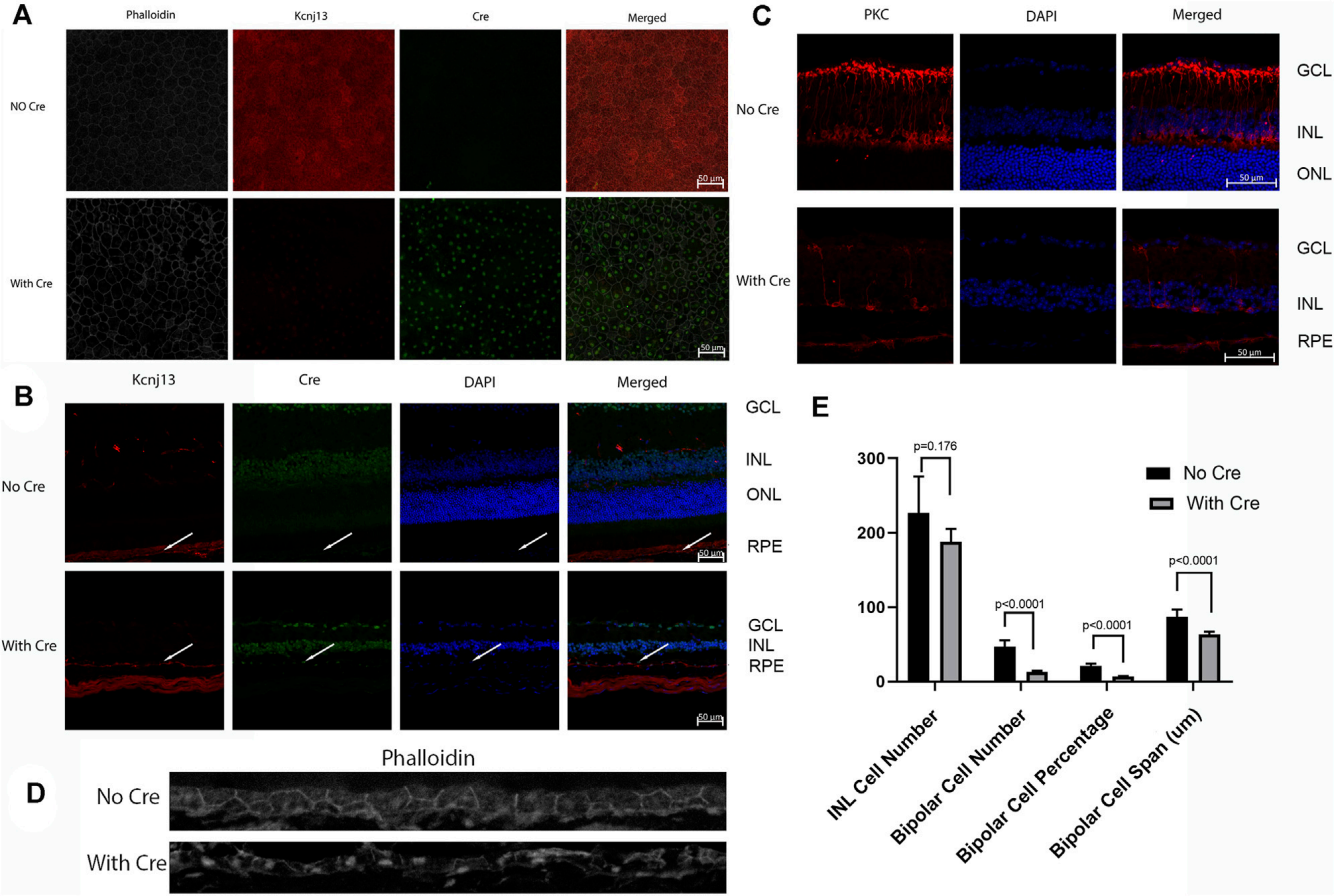
Cre and Kcnj13 expression. **(A)** RPE flatmounts of 9-month-old KCNJ13CKO ko/ko, VMD2-Cre −/− (top row) and KCNJ13CKO ko/ko, VMD2-Cre +/+ mice with high expression of Cre (bottom row). The left column shows phalloidin stained flatmounts delineating the RPE cells, the second column shows Kcnj13 expression by immunofluorescence to Kir7.1, the third column shows Cre expression by immunofluorescence, and the right column shows an overlay of the first three images. **(B)** Confocal microscopy of retinal sections from 9-month-old KCNJ13CKO ko/ko, VMD2-Cre −/− mice (top row), KCNJ13CKO ko/ko, and KCNJ13CKO ko/ko, VMD2-Cre +/+ mice with high levels of Cre expression (bottom row). The first column shows Kcnj13 expression by immunofluorescence to Kir7.1, the second column shows immunofluorescence to Cre, the third column shows nuclei by DAPI staining, and the far-right column shows an overlay of the first three images. **(C)** Confocal microscopy of retinal sections from 9-month-old KCNJ13CKO ko/ko, VMD2-Cre −/− mice (top row), and KCNJ13CKO ko/ko, VMD2-Cre +/+ mice with high levels of Cre expression (bottom row). The first column shows rod bipolar cells as shown by immunofluorescence to PKCα, the second column shows nuclei by DAPI staining, and the far-right column shows an overlay of the first two images. **(D)** Phalloidin and Cre staining of the RPE sections in 9-month-old KCNJ13CKO ko/ko, VMD2-Cre −/− mice (top row), and KCNJ13CKO ko/ko, VMD2-Cre +/+ mice with high levels of Cre expression (bottom row) showing cellular disarray and some small dense nuclei. Fluorescent channels were collected sequentially to minimize potential cross-talk between channels. **(E)** Statistical analysis of nuclei and bipolar cell number, percentage, and span (INL) in Figure 4C. Note that the decrease in bipolar cell number and INL span are greater in Figure 4B, but the statistical estimates are taken from counts distributed across the entire retina rather than a limited section.

Because the embryonic lethality in the absence of Kcnj13 prevented detailed examination of the pathological processes in the retina, the KO 1^st^ allele was converted to a conditional knockout allele by FLP recombination (Figure 1). Mice homozygous for the conditional knockout allele with LoxP sites flanking exon two were crossed with a RPE specific VMD2-Cre line (Iacovelli et al., 2011) to generate mice heterozygous for the KO 1^st^ allele. Mice of the next generation homozygous for the KO 1^st^ allele and the VMD 2 Cre allele showed Cre expression in up to 90% of RPE cells with greatly decreased or absent expression of Kir7.1 (Figures 5A,B). In control mouse retinas where no Cre is present, there is widespread expression of high levels of Kir7.1 (Figure 5A, top row). In some CKO mice Cre is expressed at intermediate levels in the RPE as estimated by fluorescence intensity (Supplementary Figure S1A) and there is spotty expression of Kir7.1, including in some cells expressing Cre. As has been reported (Keeling et al., 2020), many of the RPE cells are binucleate. In the majority of cKO mouse retinas, which showed high levels of Cre expression by fluorescence intensity, there is a complete absence of Kir7.1 expression (Figure 5A, bottom row). In the retinal sections shown in Figure 5B, while Kir7.1 staining is seen in the choroid in both the KCNJ13CKO ko/ko, VMD2-Cre −/− and KCNJ13CKO ko/ko, VMD2-Cre +/+ mice (top and bottom rows, respectively), it is only seen in the apical surface of the RPE, above the RPE nuclei indicated by arrows, in the KCNJ13CKO ko/ko, VMD2-Cre −/− mice shown in the top row. In comparison to the images in the Cre^−^ top row, there appears to be some irregularity in the size and shape of the RPE cell outlines, with occasional open areas that appear to be formed by fusion of adjacent RPE cells. These contain multiple nuclei, some of which are small and irregularly shaped, suggesting pyknosis. This is confirmed by the phalloidin and Cre stained retinal sections shown in Figure 5D, in which the RPE in the Cre^−^ retina appears as a regular array of healthy cuboidal epithelial cells. In contrast, those in the Cre^+^ retina have irregular and flattened shapes with uneven density of staining and smaller dense nuclei stained with Cre as well as condensation of the actin microfilament network.

**FIGURE 6:**
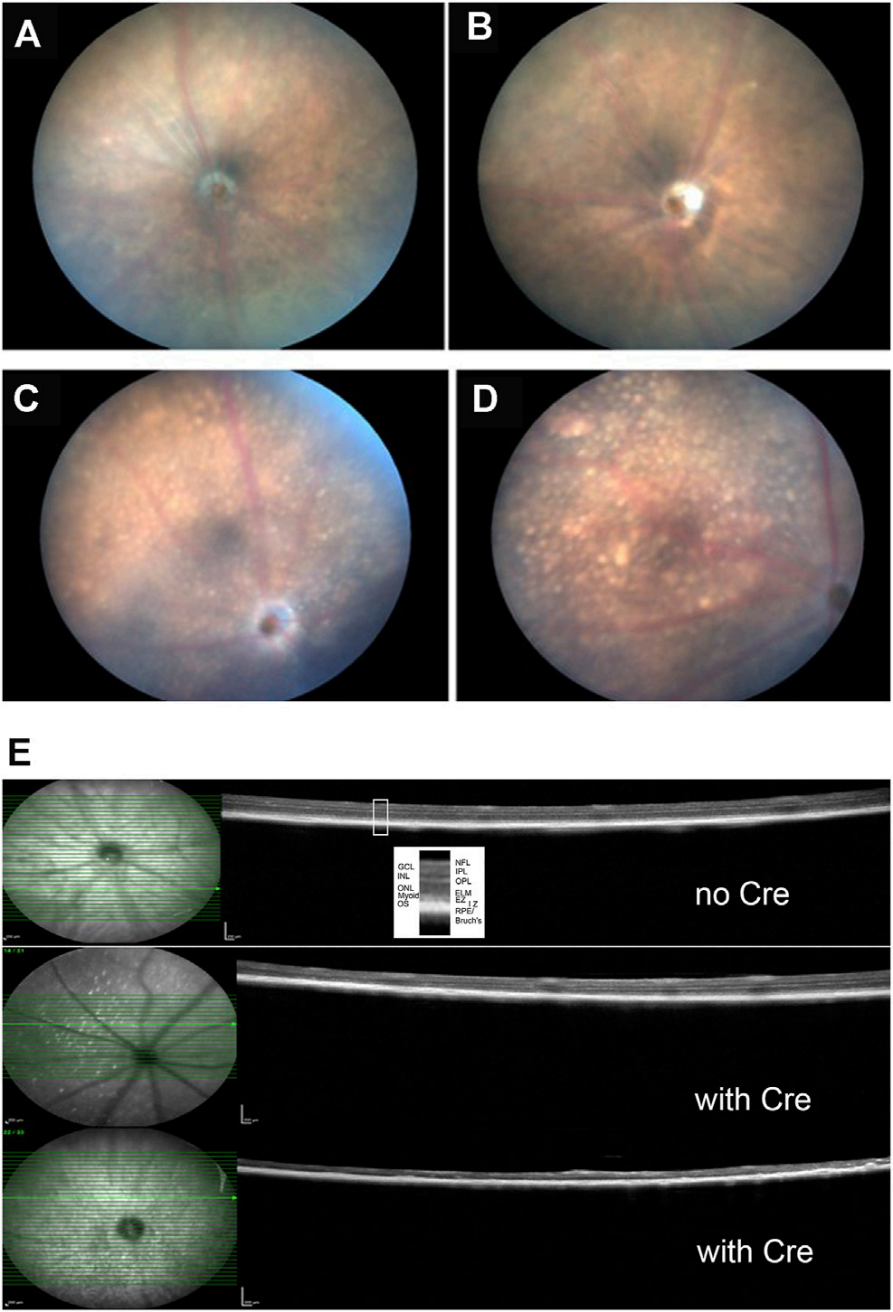
Clinical and histological findings of Kcnj13 ko mice. **(A)** representative fundus photo of 9-month-old KCNJ13CKO ko/ko, VMD2-Cre −/− mouse OD **(B)** Representative fundus photo of KCNJ13CKO ko/ko, VMD2-Cre −/− mouse OS **(C)** Representative fundus photo of 9-month-old KCNJ13CKO ko/ko, VMD2-Cre +/+ mouse OD **(D)** Typical fundus photo of KCNJ13CKO ko/ko, VMD2-Cre +/+ mouse OS **(E)** OCT results showing loss of outer retinal layers in the KCNJ13CKO ko/ko, VMD2-Cre +/+ mice. Top: month old KCNJ13CKO ko/ko, VMD2-Cre −/− mouse retinal OCT shown as a control with all retinal layers visible. Middle: moderately affected 9-month-old KCNJ13CKO ko/ko, VMD2-Cre +/+ mouse retina with an increased number of hyperreflective deposits in the NFL but with most of the retinal layers intact on the right and center but showing successive loss of the photoreceptor layers followed by the OPL in the left part of the retina. Bottom: Severely affected 9-month-old KCNJ13CKO ko/ko, VMD2-Cre +/+ retina showing loss of the outer retinal layers throughout but with preservation of the inner retinal layers including the NFL, GCL, IPL, and INL as well as the RPE and Bruch’s membrane. Increased hyperreflective deposits are also seen. Abbreviations: NFL, nerve fiber layer; GCL, ganglion cell layer; IPL, inner plexiform layer; INL, inner nuclear layer; OPL, outer plexiform layer; ONL, outer nuclear layer; ELM, external limiting membrane; Myoid, myoid component of the inner segment; EZ, ellipsoid zone of the inner segment; IZ, interdigitation zone of the outer segments and apical processes of the RPE; RPE, retinal pigmented epithelium; Bruch’s, Bruch’s membrane.

Fundus photographs of wild type and Kcnj13 cKO mice are shown in Figures 6A–D. The top two panels, A. and B., show typical retinas from wild type mice with some retinal stippling but no specks or inclusions. The bottom two panels, C. and D., show typical fundi from a Kcnj13 cKO mouse, which have distinct inclusions, especially seen in the OS, and some attenuation of the retinal vessels. In some retinas, e.g., OD in panel 5C, the optic disc appeared pale, but this was not a consistent finding.

Retinal OCTs from Cre^−^ mice show all retinal layers visible across the fundus with only a few hyperreflective foci in the nerve fiber layer (Figure 6E, top panel). The retina shown in the middle panel is from a cKO mouse with moderate Cre expression and shows an increased number of hyperreflective deposits in the NFL but with most of the retinal layers intact on the right and center. However, there is successive loss of the photoreceptor cells followed by the OPL in the left part of the retina. A more severely affected cko retina is shown in the bottom panel, having loss of the outer nuclear layer throughout but with preservation of the inner retinal layers including the NFL, GCL, IPL, and INL as well as the RPE and Bruch’s membrane. Increased hyperreflective foci are also seen not only in the NFL but also in the RPE/Bruch’s layer.

The effects of knocking out Kcnj13 on the retinal structure are shown in more detail in Figures 4E–G. Figure 4E shows an H&E-stained retina from a 9-month-old wild type mouse (Kcnj13CKO +/+, VMD2-Cre +/+) with good preservation of all retinal layers. Figure 4F shows a conditional ko (Kcnj13CKO ko/ko, VMD2-Cre +/+) retina with the retinal layers initially relatively well preserved on the left side of the optic nerve, but with more severe changes on the right and peripherally on both sides, while Figure 4G shows a conditional ko (Kcnj13CKO ko/ko, VMD2-Cre +/+) retina with severe changes throughout. The well-preserved retinal area in Figure 4F reflects a lack or lower level of Cre expression in that region (see above and Figure 5A; Supplementary Figure S1). The transition from the preserved retina immediately to the left of the optic nerve to the affected area farther left is particularly informative. Degeneration of the inner and outer segments of the photoreceptor layers can be seen, followed by loss of the outer nuclear layer. In contrast, the inner nuclear layer and the rest of the inner retina appear well preserved even in areas affected by retinal degeneration. In addition, the RPE itself is also preserved, although somewhat more widely and irregularly spaced, separated by clear lacunae and irregularly shaped hematoxylin positive material.

Confocal fluorescent microscopy of retinal sections from KCNJ13CKO ko/ko, VMD2-Cre −/− mice shows no Cre but Kir7.1 signal in the apical borders of the RPE (Figure 5B, top row, arrows), along with significant background shown by both the Cre and Kir7.1 antibodies. With Cre expression in the RPE (Figure 5B, bottom row, arrows) Kir7.1 staining in the apical borders of the RPE is decreased or absent, leaving only background staining of Bruch’s membrane confirming the effectiveness of the conditional CRE ko on Kcnj13 expression. It is also noteworthy that the Kcnj13 cKO mouse retinas have lost the photoreceptor layer and much of the OPL, and that Kcnj13 staining is not seen at the apical surfaces of the RPE as in the control retinas (arrows). Use of an anti-mouse secondary antibody to detect the Kir 7.1 mouse primary results in background labelling of choroid due to presence of mouse IgG in this vascularized layer just below the RPE nuclei. The relationship between Cre expression and the absence of Kcnj13 expression can be seen more clearly in Supplementary Figure S1B. Here, Kir7.1 staining in the apical RPE above Bruch’s membrane is evident in the control retinal sections without Cre expression shown in the upper row of panels. When Cre is expressed at high levels Kir7.1 is absent from the apical RPE (Supplementary Figure S1B, middle panels), and RPE nuclei can be seen to be expressing Cre. In contrast, retinas in which Cre expression is inconstant show patchy expression of Kir7.1 in the apical RPE (Supplementary Figure S1B, bottom panels). Here, RPE cells expressing Cre (arrows on the right and left of each panel) show no overlying apical Kcnj13 expression. In contrast, the center cell (middle arrow) does not express Cre and has patchy apical staining for Kir7.1 overlying the nucleus.

To investigate cell loss in the retina of the Kcnj13 cKO mice further, retinal sections were subjected to immunofluorescence confocal microscopy with antibody to PKCα, which serves as a marker for rod bipolar cells (RBCs) (Caminos et al., 2000). The top row of panels in Figure 5C shows the distribution of rod bipolar cells in a KCNJ13CKO ko/ko, VMD2-Cre −/− control mouse. The cell bodies occupy most of the outer row or, in some places, two rows of the inner nuclear layer, with dendritic processes extending into the outer plexiform layer and their axons extending to the inner part of the inner plexiform layer with thick club-like branches abutting the outer layer of ganglion cells. Retinas in KCNJ13CKO ko/ko, VMD2-Cre +/+ mice show much less PKCα staining (Figure 5C, bottom row). As shown in the H&E-stained sections (Figures 4E,F), the OCT (Figure 6E), and retinal confocal sections (Figure 5B), the entire photoreceptor is absent, so that the inner nuclear layer abuts the RPE, which is seen as a single row of nuclei in the DAPI stained middle panel with some PKCα positivity, possibly background, in the choroid. There are only scattered cell bodies showing PKCα reactivity in the top row of cells of the inner nuclear layer, which as in Figure 5B, has the suggestion of being thinned and contain fewer nuclei, although this is not statistically significant in this section (Figure 5E). However, there are significant decreases in the number and percentage of bipolar cells as well as their span. These cells show no dendritic processes, and their axons are attenuated, showing fewer terminal branches, which also appear thinned. In addition, as it has been reported that zebrafish with the obdtd15 Kcnj13 mutation shows alterations in phagosomal clearance and mitochondrial area (Toms et al., 2019), mitochondrial staining with ATP5a in wt and Kcnj13 conditional ko mice was compared (Supplementary Figure S2). While no difference was observed in the density of staining between the ko and wt mice, there was a significant loss of length of the photoreceptor inner segments, both in absolute length and relative to the outer segments, which were relatively well preserved until late in the degenerative process. In addition, even with intermediate levels of Cre expression, shortening of the photoreceptor inner segments was accompanied by loss of photoreceptor morphology as seen in the middle row of Supplementary Figure S2A and progressively decreased thickness of the outer nuclear layer as shown in Supplementary Figure S2A and presented graphically in Supplementary Figure S2B. Similarly, no obvious differences were noted in the density of RPE phagosomes or mitochondria on EM (data not shown). These results are consistent with those seen in human iPSC-RPE cells (Shahi et al., 2019). These findings were equally present in male and female mice.

**FIGURE 7:**
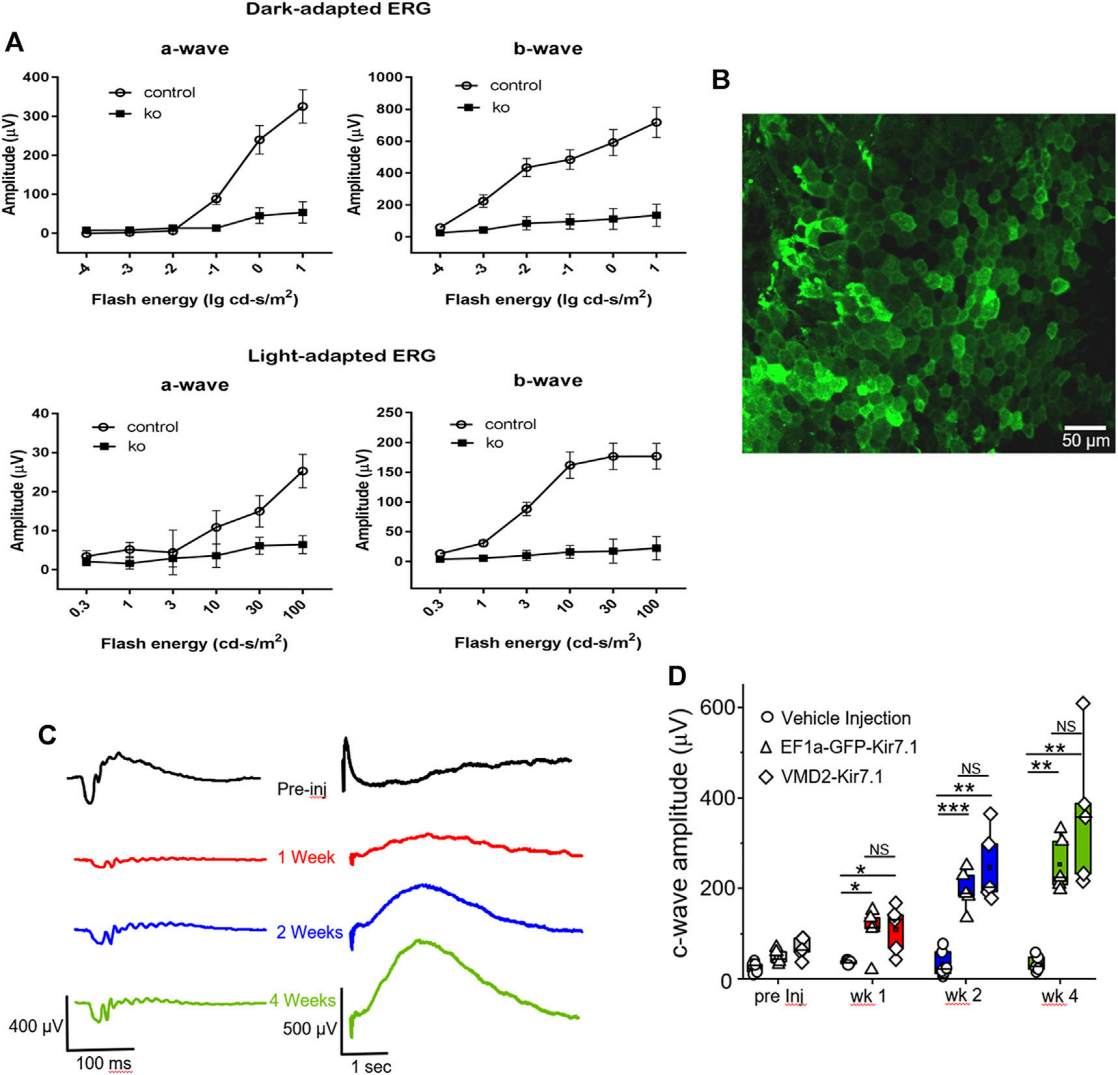
Functional assessment and gene therapy of Kcnj13 ko mice. **(A)** Scotopic (top) and photopic (bottom) a-wave (left) and b-wave (right) ERG amplitudes of 6-month-old KCNJ13CKO ko/ko, VMD2-Cre +/+ (10 mice) and KCNJ13CKO ko/ko, VMD2-Cre +/+ (10 mice) mice. **(B)** Lentiviral transduction of RPE cells rescues the c-wave in the KCNJ13CKO ko/ko, VMD2-Cre +/+ knock out mice. Representative image of the RPE flat mount showing membrane localization of the Kir7.1 protein tagged with GFP 4 weeks after lentiviral transduction. **(C)** Representative ERG traces from KCNJ13CKO ko/ko, VMD2-Cre +/+ mice showing a-, b- and c-wave during pre-injection, week 1, week 2, and week 4 after lentiviral gene augmentation.**(D)** Comparison of the average plots of c-wave amplitude during the course of lentiviral transduction of RPE cells expressing Kir7.1 driven by either EF1a or VMD2 promoters or vehicle. NS: Not Significant; *p < 0.05; **p < 0.005; ***p < 0.0005.

The morphological signs of retinal degeneration were accompanied by loss of visual function as shown in Figure 7A, which shows the a- and b-wave amplitudes of scotopic and photopic ERGs from Kcnj13 cKO Cre^+^ and Cre^−^ mice as they vary with flash energy. Both the scotopic and photopic Kcnj13 cKO Cre^+^ ERG a- and b-waves are extinguished, consistent with the loss of the entire photoreceptor layer shown in Figures 4F,G, 5B,C, as well as the retinitis pigmentosa phenotype seen in humans with loss of function KCNJ13 mutations, or in some cases highly attenuated, consistent with the patchy degeneration seen in a relatively small group or mice (Supplementary Figure S1). Furthermore, when scotopic ERG was performed on Kcnj13 cKO Cre + mice, the amplitude of the c-waves that originate from the RPE cells was severely reduced (Figure 7C preinjected). When gene replacement therapy is carried out by subretinal delivery of lentivirus carrying the GFP-tagged Kir7.1 gene driven by either the EF1a or VMD2 promoters, GFP expression was observed in the RPE cells at 4 weeks after surgery (Figure 7B). The morphology of the RPE cells was preserved, and strong GFP fluorescence was observed with the Kir7.1 protein being trafficked to the membrane. The amplitude of the c-wave in mice transduced with lentivirus containing the KCNJ13 gene increased significantly after 2 weeks (Figures 7C,D), reaching roughly 5 times the pre-injection level by 4 weeks after injection, driven by either the EF1a (*p* < 0.005) or VMD2 (*p* < 0.005) promoters. Unlike the eyes injected with the virus, the eyes injected with the vehicle, as a control, had no change in the c-wave during any of the tested time points. (Figures 7C,D). Throughout this time period, the a- and b-waves show little increase, consistent with the complete degeneration of the photoreceptors seen in the histology and confocal microscopy results.

## Discussion

Here we investigated the implications of a lack of Kir7.1 function on the retina in a mouse conditional knockout model. Initially an unconditional knockout in which *β*-galactosidase was expressed from the Kcnj13 promoter was used, but no live embryos were detected after embryonic day 15, strongly suggesting that absence of Kir7.1 is an embryonic lethal in this strain, although other Kcnj13 knockout mice have been reported to survive to birth (Zhong et al., 2015; Yin et al., 2018). The importance of Kir7.1 for embryonic development is also emphasized by the smaller size of the homozygous Kcnj13 KO relative to heterozygous or wild type embryos. However, the relative importance of Kcnj13 expression for the eye is demonstrated by the dense *β*-galactosidase staining in the embryonic eye relative to the remainder of the embryo. Embryonic expression of Kcnj13 is restricted to the anterior parts of both the neural retina and RPE, while it is expressed not only in the RPE, but also in the inner nuclear layer, nerve fiber layer, inner plexiform layer intensely in the internal limiting membrane in human adults (Hejtmancik et al., 2008). It is interesting that the region of high embryonic Kcnj13 expression will later form the ciliary body, which might explain the vitreal degeneration seen in snowflake vitreoretinal degeneration patients. Similarly, expression in the embryonic lens might also correlate with all SVD and KCNJ13 related arRP patients developing cataracts by early adulthood. The higher level of staining in central lens fiber cells relative to the anterior epithelial cells of the lens might reflect the amount of time over which the *β*-galactosidase protein has been accumulating as the epithelia differentiate into fiber cells, as Kcnj13 mRNA levels are higher in the epithelia than fibers in both the mouse and chick (Hawse et al., 2005; Hoang et al., 2014).

In conditional knockout mouse retinas, Kcnj13 expression in the RPE is expected to be eliminated by Cre expression. However, this correlation is not perfect, and in some retinas a few RPE cells expressing Cre also show residual Kcnj13 expression, presumably due to lower than threshold level of Cre expression in these cells. This is consistent with preservation of reduced ERG expression in a subset of KCNJ13CKO ko/ko, VMD2-Cre +/+ mice. In addition, it has been shown recently that Cre expression with this model shows some leakiness with expression in other retinal cells than the RPE (Chen et al., 2021; Choi et al., 2021). This is also apparent in Figure 5B, in which Cre expression is seen not only in the RPE but also the GCL and INL. However, as Kcnj13 is not expressed in those cells, as also shown in Figure 5B, this should not affect the results seen in this mouse model.

Overall, however, lack of Kcnj13 expression in the overlying RPE caused degeneration of the subjacent photoreceptors. This was accompanied by loss not only of the outer nuclear layer, but also the outer plexiform layer as shown by the loss of dendritic processes of rod bipolar cells. This loss of the outer retinal structures with relative preservation of the inner retina is seen on OCT and recapitulates, to some degree, that seen in human LCA resulting from a homozygous c.722T > C p.(Leu241-Pro]) KCNJ13 mutation (Sergouniotis et al., 2011). The inner nuclear layer also is thinned and shows loss of most of the bipolar cell bodies. Those rod bipolar cells that remain not only have lost their dendritic processes, but their axons also appear to be degenerating, with loss of their normal morphology. While the original insult to the photoreceptors certainly results from the failure to maintain potassium homeostasis in the subretinal space (Shahi et al., 2017), the damage is probably compounded by the general degeneration of the outer retina resulting in toxic debris secondary to the photoreceptor death. That this is the case is also suggested by the toxic changes evident in the RPE cells themselves. Given the extensive damage done to the outer retina, it is not surprising that rescue of Kcnj13 expression in the RPE is not accompanied by complete recovery of the a- and b-wave amplitudes, but does show recovery of the c-wave, which is generated by the RPE, at least in rodents (Perlman et al., 1995).

A comparable retinal degeneration model is the RCS rat, in which the RPE is unable to phagocytize rod outer segments as they are shed, due to a mutant Mertk causes photoreceptor degeneration with preservation of the gross structure of the inner retina (Ball et al., 2003). However, the inner retina does show reactive changes in Müller cells and altered branching patterns in bipolar and horizontal cells. However, cone bipolar cells in this model show increased expression of recoverin with an overall normal organization but atrophy of rod bipolar cell terminals but preservation of cell numbers and axonal projections. These differences might relate to the more chronic and progressive nature of retinal degeneration in the RCS rat relative to that in the Kcn13 cKO mouse and expression of PKCα in rod but not cone bipolar cells (Pan, 2000).

Thus, these results show that while the inwardly rectifying potassium channel Kcnj13 is responsible for maintaining potassium and ionic balance across the RPE, the importance of this goes well beyond facilitating light stimulated discharge of the photoreceptors and is necessary for their survival. In this sense, the downstream pathophysiology of Kcnj13 based retinal degenerations is similar to those of other causes of retinitis pigmentosa, causing photoreceptor or RPE cellular damage and death more directly. While this conditional knockout mouse as a loss of function mutation models autosomal recessive retinitis pigmentosa (Sergouniotis et al., 2011; Pattnaik et al., 2015), it seems likely that the downstream pathophysiology of the c.484C > T, p.(R162W) mutation implicated in snowflake vitreoretinal degeneration might be similar (Hejtmancik et al., 2008). However, that mutation results in depolarization of the resting membrane potential (Hejtmancik et al., 2008; Pattnaik et al., 2015) and might also act in a dominant-negative fashion to inhibit normal Kir7.1 channel function at least partially, either or both of which could explain the dominant inheritance pattern of snowflake vitreoretinal dystrophy (Zhang et al., 2013; Pattnaik et al., 2015). While it will require additional detailed future studies, it seems possible that partial preservation of the inwardly rectifying potassium channel activity in heterozygotes with the R162W change might contribute to the somewhat milder retinal changes in SVD, while depolarization of the resting membrane potential might be responsible for the vitreous changes, which are not seen to this degree in the arRP and LCA cases (Lee et al., 2003; Sergouniotis et al., 2011; Perez-Roustit et al., 2017).

In conclusion, we have used a conditional Kcnj13 knockout mouse model to explore the retinal pathophysiology of KCNJ13 based retinal degenerations, confirming and extending previous observations. Early embryonic expression patterns of Kcnj13 fit well with the characteristics of SVD, LCA, and arRP resulting from mutations in KCNJ13. While the initial insult of these mutations is the documented loss of inwardly rectifying potassium channel activity, coupled in the case of SVD with the leakiness of the mutant Kir7.1 channel, the final pathology appears to be the death of the retinal photoreceptors with attendant degeneration of the entire outer retina, similar to that of other many retinal degenerations.

## Data Availability

The original contributions presented in the study are included in the article/Supplementary Material, further inquiries can be directed to the corresponding author.

## Supplementary Material

Supplementary Figure S1 Inverse relationship between Cre and Kcnj13 expression. **(A)** RPE flatmounts of 9-month-old KCNJ13CKO ko/ko, VMD2-Cre −/− (top row) and KCNJ13CKO ko/ko, VMD2-Cre +/+ mice with spotty intermediate expression of Cre (bottom row). The left column shows phalloidin stained flatmounts delineating the RPE cells, the second column shows Kcnj13 expression by immunofluorescence to Kir7.1, the third column shows Cre expression by immunofluorescence, and the right column shows an overlay of the first three images. Note that Kcnj13 expression occurs in some RPE cells expressing Cre, but it tends to be at lower levels than those in KCNJ13CKO ko/ko, VMD2-Cre −/− mice. **(B)** Confocal microscopy of retinal sections from 9-month-old KCNJ13CKO ko/ko, VMD2-Cre −/− (top row), KCNJ13CKO ko/ko, VMD2-Cre +/+ mice with high levels of Cre expression (middle row), and KCNJ13CKO ko/ko, VMD2-Cre +/+ mice with spotty Cre expression. Note the absence of Kcnj13 staining in the basal surface of the RPE (above the nuclei) in the middle row absence of Kcnj13 staining in the basal surface of the RPE (above the nuclei) in the middle row and the presence of Kcnj13 staining above the middle nucleus (DAPI but not Cre positive) and the absence of Kcnj13 staining at the base of both flanking cells with Cre stained nuclei. Arrows point to RPE basal surfaces above nuclei while *indicates the basal surface of the RPE and + indicates the choroid.

Supplementary Figure S2 Confocal microscopy of mitochondrial density in retinal sections from 9-month-old KCNJ13CKO ko/ko, VMD2-Cre −/− mice (top row), KCNJ13CKO ko/ko, VMD2-Cre +/+ mice with intermediate Cre expression (middle row), and KCNJ13CKO ko/ko, VMD2-Cre +/+ mice with high levels of Cre expression (bottom row). The first column shows ATP5A expression by immunofluorescence, the second column shows DAPI staining of nuclei, and the third column shows an overlay of the first 2 images. White lines indicate measurements of the thickness of the inner segment of the photoreceptor layer as indicated by the presence of mitochondria and purple lines indicate the thickness of the ONL. Measurements were taken from similar regions of the retina from 5 KCNJ13CKO ko/ko, VMD2-Cre −/− mice, 5 KCNJ13CKO ko/ko, 5 VMD2-Cre +/+ mice with intermediate Cre expression (middle row), and 5 KCNJ13CKO ko/ko, VMD2-Cre +/+ mice with high levels of Cre expression. **(B)** Length of the photoreceptor inner segment (left) and outer nuclear layer (right). The ANOVA f-ratio value is 53.3 (*p* < 10^–6)^. Pairwise comparisons are by Tukey’s HSD test.
