# Supplementary figures and images for: Retinal Development and Pathophysiology in Kcnj13 Knockout Mice
Source: Front Cell Dev Biol. 2022 Jan 12;9:810020. doi: 10.3389/fcell.2021.810020 (PMC8790323; doi:10.3389/fcell.2021.810020)

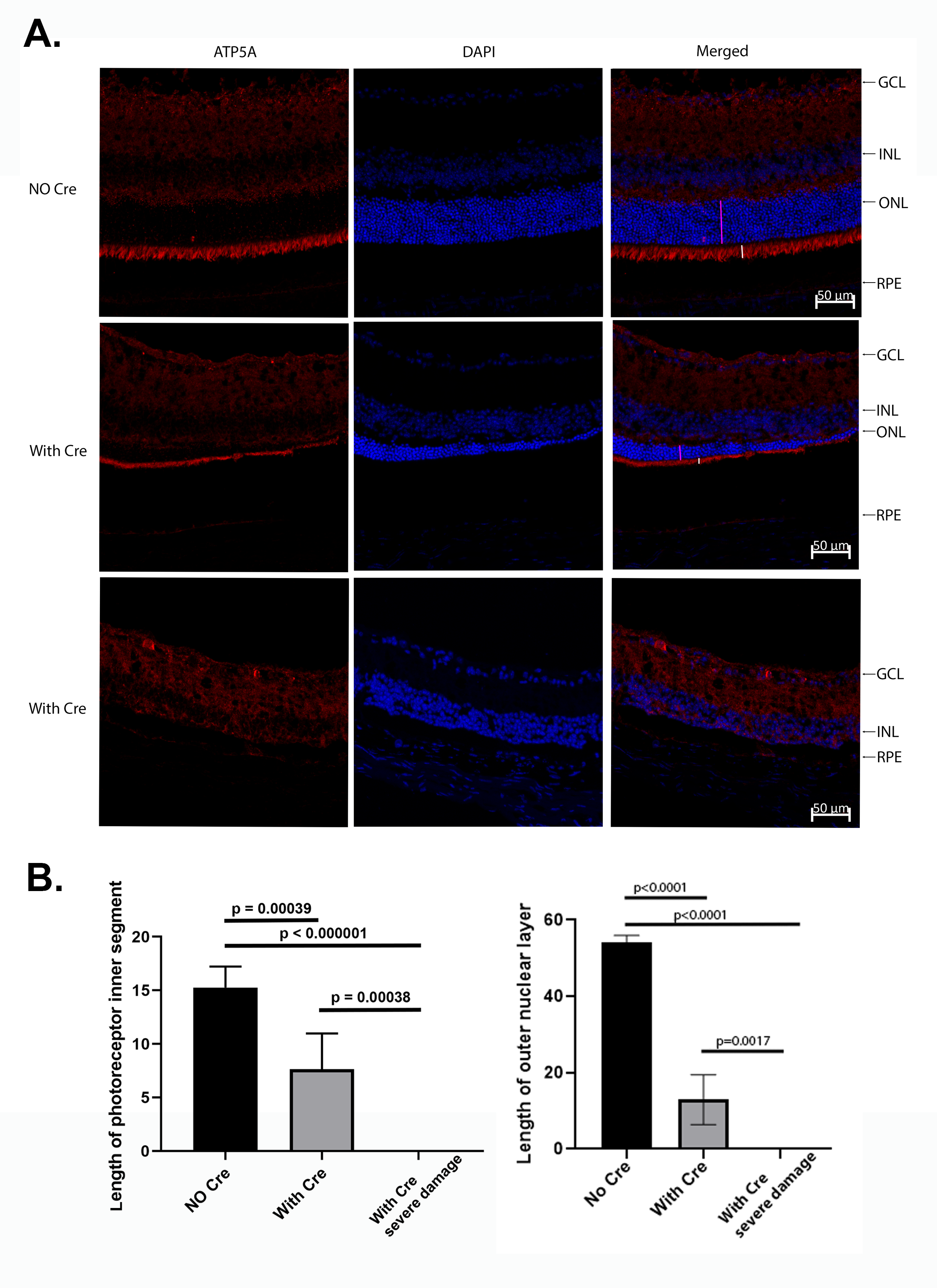

Supplement: Supplementary file 1 [file Image2.tif]

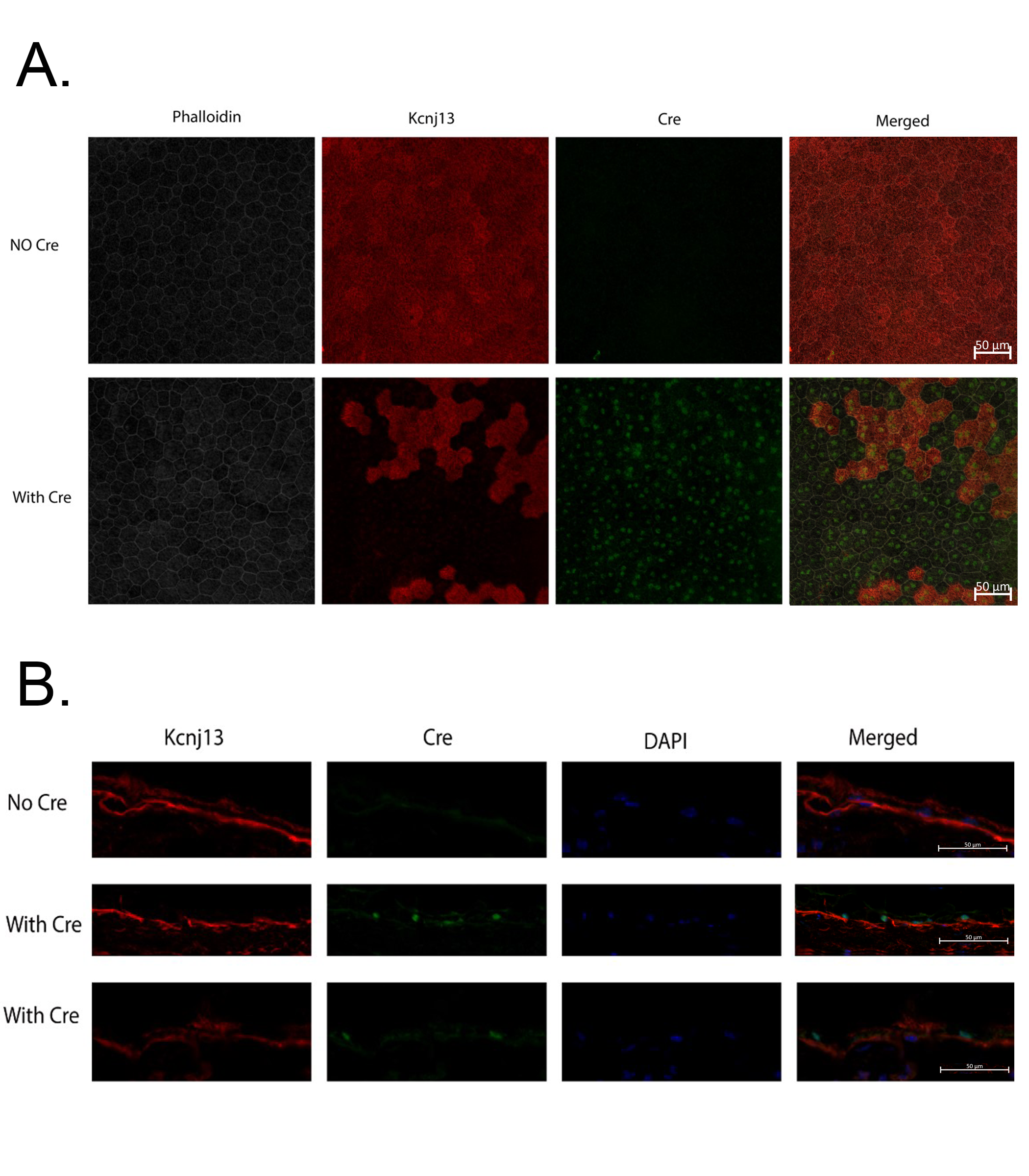

Supplement: Supplementary file 2 [file Image1.tif]
